# Dental Operations That Are a Menace to the Teeth and Surrounding Tissues

**Published:** 1910-08-15

**Authors:** W. H. Whitslar

**Affiliations:** Cleveland, Ohio


### DENTAL OPERATIONS THAT ARE A MENACE TO THE TEETH AND SURROUNDING TISSUES.

---

W. H. Whitslar, m.d., d.d.s., Cleveland, Ohio.

---

It is my desire to impress the fact that many of the faults of bad operations upon the teeth are due to poor diagnosis, which further leads to bad judgment, for, the one is founded upon the other. In this way we can see the possibility of entirely wrong operations being performed.

Example.—There are times, rarely however, when the first permanent molars should be extracted. These are difficult cases to determine, and the temptation to extract this molar when broken down is powerful. To forsee the results of malocclusion in a case of extraction is not only good diagnosis, but rare judgment, if future disaster is to be prevented. Many examples could be given, wherein dependence upon diagnosis is all important.

Having made a diagnosis, the greatest question one can submit to himself is, "Will this operation restore these tissues to a normal condition, whereby their function is made the best?"

What may be a natural condition in a mouth, may not be a normal or typical condition of the teeth and surrounding tissues. In such a case improvement may be prescribed. Observe a mouth in which the dental arch is irregular. The subject apparently lives well, the dental arch is natural to him, but it is not normal. Nature established his condition imperfectly, due to some hereditary conditions, or all too frequently, because of improper extraction, or retention of the teeth. In this the standard or rule of the development of the dental arch has been altered, and the symmetry is lacking. To normalize this condition the orthodontist predetermines the results of applying forces to this and that condition ere the teeth and jaws are brought into their normal occlusion. In performance of these operations he may make errors of adjusting these forces. He may use poorly constructed or ill-fitting appliances, which retain food, causing decay of the teeth and irritations of the gingivae, cheeks or tongue. Too rapid movement of the teeth may cause pericementitis and even death of the dental pulp. The application of force in the wrong direction, or improper retention of the teeth are detrimental to the teeth and surrounding tissues. Constant movement of the teeth does not allow nature to follow with depositions of lime-salts sufficient to keep up the equilibrium. More serious than all these is the possibility of disturbance of digestion, neurasthenia, and even nervous prostration to be considered. Orthodontia is a serious operation to subject some children to, for no doubt cases occur which require the keenest insight to the welfare of the child. Insanity has been produced by orthodontia operations.

In the question of diagnosis, the dental specialist, like all other specialists, is ever subject to having his judgment warped in favor of his own specialty. The specialist perceives more clearly the conditions pertaining to the line of study he is engaged in, and ordaines their superiority over others. In consequence of this, his judgment may suffer. The crown and bridge specialist might offer his particular service, the prosthetist would desire an artificial denture, and the one interested in the treatment of pyorrhea alveolaris advise saving teeth, when extraction is desirable.

Are people safer in the hands of a very intelligent all around dentist? My answer is, "Yes," because he is able to predict failure for himself if he undertakes the work of a specialist in which he knows he cannot be successful. He knows his safe course is a to refer the case to a specialist and thus safeguard his own reputation. This decision in the first place, was based upon his diagnosis, and its value cannot be over-estimated.

I have thus emphasized the first importance of diagnosis to lessen the possibility of menacing the teeth by improper operations.

The hereditary conditions of the mouth and teeth may preclude the possibility of doing any service which does not have some detrimental influence to the teeth. Likewise diseased teeth frequently are beyond the pale of comparitive restoration. This is because there are no reparative tissues to utilize, as in general surgery.

Dentistry performs restorative and constructive service unequaled by the surgeon frequently. While the reperation is mechanical, it is built upon a *living base*, which constitutes the operation *surgical science*.

Dentistry has for its object the conservation of the teeth in order to masticate food properly, to assist in speaking, etc. Every operation that falls short of assisting these functions is detrimental and a menace to health.

The operations that may be a menace to the teeth and surrounding tissues are many, but it may be said that the fault lies mostly with the technical skill in their execution.

The beauty of practicing dentistry is the variety of study it affords, giving food for scientific thought and decision.

Every condition in the mouth is corelated to some other, hence when deciding what is best for one tooth, its relations with the adjoining and opposing teeth are necessary. If a filling is to be inserted, the kind of filling, if any, in the next tooth is to be noted so as to have similarity of materials both as to color and physical qualities. Durability and strength of filling materials are given precedence over color or esthetic effects in most all operations. When teeth are beyond filling the question of extraction, or using them as foundations for artificial teeth becomes momentous. Judgment dictates the removal of diseased teeth and adjacent tissues seriously affected by abscess or purulent discharges that are not amenable to treatment. The retention of such conditions serve as irritants to the surrounding tissues and also to the nervous system, which may result in neuritis, neurasthenia, and even insanity.

It is complimentary to the dental profession that a medical insanity expert should make exhaustive studies to learn the possibilities and certainty of many cases of mental disease being instigated by the teeth. Dr. W. S. Upson, of Cleveland, Ohio, is thus contributing to medical science valuable data. From these we are taught that every means should be taken by dental practitioners to eliminate abscessed conditions, also impacted and some unerupted teeth. The skiagraph is the principal means of arriving at a correct diagnosis in these cases, and should be used more frequently in dentistry.

Formerly the wholesale extraction of teeth was considered the greatest offense in dentistry. In 1879, a noted writer, Dr. J. W. White, stated that there were twenty millions of teeth extracted annually in the United States. For a time it would seem that this number increased, as we observed the large exhibits of extracted teeth before so-called "Dental Parlors." These exhibits are disappearing, and now many "snags" are utilized for the wonderful discovery of the age, the "Alveolar Method." This is another misnomer that has crept into puplicity. It is a species of graft applied to dead and decayed timber, (teeth), whose only sprouts are dollars poured into the pockets of pretenders.

It is often a serious question to decide whether it is better to extract teeth and advise a denture, or retain some, and use either a partial denture or a bridge. Strong teeth are necessary for a partial plate or a bridge. Both methods are a menace to the teeth and tissues supporting them, but this may be condoned if a reasonable service for a considerable time is certain. The pros and cons of the situation should be explained to the patient and his preferences weighed.

Bridgework properly constructed is one of the greatest blessings to humanity, but sometimes the foulest mouths are those containing it. I have seen fractured jaw-bones held firmly and mastication made perfect by bridgework. It is known to have caused extreme nervous debility and insanity. The covering of roots of teeth under a bridge is reprehensible, and filthy practice, if the bridge is not attached to them.

The greatest menace to the teeth by crowns and bridgework is the effect upon the gingivae. The adaptation of this work to the piers is difficult and often is mismanaged, because the gingivae are not measured before bands are fitted. By carefully examining the gingival margins with a probe, it will give a proper idea of the distance under the border a band may be made to extend. Unless the fitting is perfect the edges become irritants and the beginning of all forms of inflammations are likely to occur and destruction of the tissues follow. This begins as a traumatic disturbance of the dental ligament. When this is destroyed an undue strain is placed upon the principal fibres of the peridental membrane, which from overstrain becomes neurasthenic and a passive condition obtains. From this it is only a step to interstitial gingivitis and a general break bown of the membrane. The difficulties arising from these causes is a story in itself.

I have no doubt that many bridges are inserted when a denture would be better in every way Even these will distort and irritate the tissues at times, to say nothing of the inartistic arrangement of the teeth.

Dr. J. Leo Williams, who is now perfecting models for artificial teeth, will revolutionize many of the staid forms of teeth and awaken the average dentist to the greatest possibilities of artistic, as well as the practical construction of dentures. This anatomical perfection will insure greater masticatory power and conserve the tissues.

It should have been mentioned that in the preparation for bridgework or crowns, splitting of roots, is a serious defect. Fracturing both roots and alveolus in extracting teeth is productive of much pain and after effects, especially if impure hypodermatic injections are used.

One of the greatest boons to humanity is the preservation of the teeth by filling cavities produced by decay. Dangers from these operations arise in the excavation of the cavities, near approach to the pulp, capping of pulps, and unusual destruction of tooth structure. Or, if the tooth pulp dies, then its removal and filling of the canal constitutes an operation that may forebode the greatest troubles that arise in dentistry. The excitations produced by clamps, ligatures, separators, separating the teeth, and filling materials, are comparable to the imperfect results of root fillings. Opening up canals, drilling through the walls, allowing fillings to protrude into the apical space, suggests the neuclus of direful disturbances. To enter into a discussion of this phase of our subject would require many pages to describe it fully.

Dr. I. N. Broomell has shown the effect of large masses of gold to be harmful to a living tooth. For instance, there may be a disintegration of the enamel in connection with cement, which disintegrates beneath masses of gold. Also, diseases of the pulp will occur, which result in their death. No one should fail to read Dr. Broomell's article published in the Dental Cosmos for April, 1910. Each one of the above factors could excite a discussion as to their demerits pertaining to filling operations and materials.

In completing a filling one of the difficulties is to finish it so as to have a normal contour, and not disturb the tissues at the cervical margin of the tooth. The contour should be such as to extend beyond all points of contact and to serve as an incline for food to naturally fall into the cavity of the mouth. By contour, also service is rendered to obliterate food spaces between the teeth. These spaces invite disease of the gingivae and peridental membrane. Careless separation of the teeth, will also promote the same difficulties. The preservation of the gingivae from accidental or careless operating is second only to the filling of carious teeth. The preservation of the interdental spaces is of greatest importance. What is true of fillings is also true of inlays, either porcelain or gold. The ideals are the same, but there may be a tendency to allow the filling in the crevical part to go without careful reducing to the exact edge of the cavity. The inlay properly constructed usually demands more sacrifice of the tooth structure than fillings inserted in the old way, dut they are less likely to damage the pulp or gingival tissues. Inlay filling has taught clearly the importance of "extension for prevention."

There are few medicaments used in dentistry that are harmful. However, it is well to state that destruction of the dental pulp is sometimes accompanied by pericementitis or even destruction of the membrane unless great care is observed in the use of arsenious acid. Nitrate of silver, concentrated solutions of hydrogen dioxode, zinc chlorid, phenol, and sulphuric acid, are other caustics that may be used detrimentally to the teeth and tissues. Discolorations of the teeth due to nitrate of silver are produced by forming albuminate of silver, which acts as a resisting force to decay. Oil of Cassia used in pulpless teeth discolors, because of furfurol, a chemical, which turnes brown on exposure to air and light. While cocain is a protoplasmic poison when brought into contact with living tissue, its specific action is utilized in producing paralysis of nerve endings in dentistry. Solutions of cocain should be made at the time of their use. Ready-made solutions are dangerous, because of their decomposition after exposure to air. Careless injections of cocain may produce inflammations of the mucous membrane, and also be contaminated by the poisons of specific disease. Dr. Herman Prinz has recorded in his Dental Materia Medica and Therapeutics very interesting cases of psychic disturbances by injections of cocain.

The conclusion of this paper would not be complete without reference to the subject of so-called "Cleaning of teeth." Let us dignify it as an "operation," by letting it have the merit of calling forth the finest manipulations possible. It is not an ordinary procedure, though treated as such by many. It has a range of removing microscopic plaques to that of calculi as large as a hen's egg. With a perfect operation it promises immunity from decay and recovery from pericemental diseases, due to extraneous substances. Careless, thoughtless, and unskilled manipulation with improper instruments induce disease, leaving injury and filth everywhere. Microbic nests are in every corner awaiting conditions to propagate larger colonies. Dental decay may run riot, the gingivae ulcerate, dental ligament melt away and the fibres of the membrane lose their grip upon the alveolus and the tooth is forever lost.

One of the greatest mistakes dentists have made in the past, has been the slight attention given to the removal of all foreign materials from the surfaces of the teeth in a thorough manner, and polishing these surfaces afterwards.

It is not only fair but just that a great tribute should be given to Dr. D. D. Smith, of Philadelphia, who has pleaded, urged and demonstrated to the profession, the great value of treatment, now called "oral prophylaxis." With such ideals of treatment consummated and thorough mastication of food mastered, may we not look forward to that condition of the perfect man.

### DISCUSSION.

Dr. Chas. Morrison: The paper which we have just listened to, it appears to me, is one which does not allow of much discussion. The reason of this is, that there are no definite unproved theories advanced, but it is simply a resume of simple facts, which we, as practicing dentists, should all know. After reading the paper a couple of times it struck me that possibly these simple facts were just what were needed, as there is no doubt a great lack of care exhibited in many of the dental operations of the present day. This was probably the object for which the paper was written, abnd we congratulate the essayist.

It is well that the profession should constantly be kept in mind of the fact, that a dental operation, performed carelessly is almost certain to be a menace to some of the tissues of the mouth, and this paper should therefor be taken as a very potent medicinal dose by all present. If the disease is not present, then the medicine can do no harm.

In all the walks of life the man who does his work conscientiously and intelligently will have his satisfactory results, while the careless man is bound to have frequent fails ures. And these failures on the part of careless operators it must be remembered are not always due to lack of ability, but in many cases can be attributed to the fact that he tries to accomplish more work in a given time than it is possible for him to do.

In these days of progress, as applied to our profession, we are too apt to be carried away by the new ideas and methods that are from time to time being put before us. These ideas and methods would seem to enable us to get through more work, obtain good results and with less cost to our physical energy. I do not disparage the new methods, but is it not true that in many cases our patients would be much better off with the more simple operations, carefully performed? Probably, the most simple operation we have to perform is the insertion of an amalgam filling. The color of the material does not blend with the color of the normal tooth, and yet a compound amalgam filling, well contoured and finished is a work of art, and in most cases has permanent results. Such a filling should never be inserted without the use of the matrix, and should always be polished at a subsequent sitting. This would in many cases take considerable time, and the dentist, basing his charges on so much per filling, finds that the fee is inadequate.

Caps are fitted, and crowns and bridges are adjusted, and cemented in a hurried and slipshod manner, and neither the patient nor the practitioner can have the maximum amount of satisfaction with the result. The solution of the difficulty is simple; charges should be based on the amount of time required to complete the operation.

The average patient of a man whose practice is among the better class, wants good work done, and if the situation is explained to them it will not be difficult to obtain adequate fees. When a man knows that he is being well paid, it is only natural that he will take pains with his work, because he can afford to.

If the operation is along the lines of bridge-work, for example, he should proceed carefully in the preparation of his abutments for the reception of the caps, or crowns, as the case may be, step by step until the bridge is completed and put in such a way that there can be no injury to the tissues of the mouth. In many cases in injuries due to badly fitting caps, bridges and regulating appliances, protruding, and poorly finished fillings, the too violent separating of teeth, etc., etc., are limited to the extent of chronic inflammation of the gingivae and surrounding tissues, but every once in a while we find that the injury is more serious, and a malignant condition is set up, which may compel a major operation involving the loss of teeth and much bone tissue to effect a cure, if a cure is possible.

As the essayist says there are innumerable operations, which are a menace to the tissues of the mouth, I would go farther, and say, that almost every operation which we are called upon to perform, if not done carefully is likely to cause injury.

Dr. Chas. E. Pearson: Let me preface my discussion of the essay with this statement, that from over ten years careful study of the hard and soft tissues, I sm convinced that not 5 per cent. of the cases of pyorrhea, caries or necrosis of the bone are due to, and can be traced to faulty dental operations. I am firmly of the opiuion that negligence and the various predisposing influences play a greater part in the development of such conditions, than poor dentistry. I just mention this in case some of you are feeling like joining the ranks of the real estate brokers, or go out to have a horn or two.

Apart from this, I will admit that the picture of dire disaster which our essayist has so ably painted for us, opens a field for profitable discussion. The essay is admirable, inasmuch as it does not pretend to deal with the accidents which may befall the practitioner, but rather with unfortunate conditions directly following a deliberate act; which act was the result of bad judgment

For instance, a young man aged 25 years, presented himself. On the lower left side the first and second molars had been lost. The remaining teeth were in good average condition. A bridge was decided upon from the lower left second bicuspid filling in the distal surface which was removed in preparation for an inlay with a post for an abutment. The pulp was not exposed but was devitalized with arsenic. By accident the patient did not return for six days, when the tooth was very sore to pressure and particularly sensitive to heat and cold. The pulp was removed, using ethel-chloride, and a treatment of carbolic acid, sealed in for two days, it was then followed by oil of cloves. The tooth was sore and very loose. In about three weeks time, the soreness left it sufficiently to finish the preparation for inlay. A bridge was made and put in temporarily as a stay to the tooth, which is still very loose and slightly sensitive to pressure after two months. Apex large, subsequent an extraction of roots on other side of mouth showed abnormally large apical foramena.

To my way of thinking, this is a splendid case in point. Even though the apical foramen were normal, leaving no unforseen danger to arsenical poisoning, there was no necessity for the removal of the pulp, as a simple inlay with a groove into which rests an uncemented bar from the bridge, is sufficient to sustain the strain required in mastication.

That second bicuspid will never regain its normal masticating power while the bridge adds twice or more than twice the function of it. Why take such chances?

I have made it a rule never to devitalize for a bridge abutment, unless the crown portion of the tooth is weak and the pulp canal is required for the purpose of holding a post to supplement the strength of the dentine remaining in the crown portion of that tooth. Of course, it goes without saying! this only applies in cases of healthy pulp. By following this rule there is less liability to perform dental operations, which are a menace to the hard and soft tissues.

Another condition I wish to touch upon is what I would call the lazy dentist's habit, or better still the "phosphate habit of the putty dentist." It's easy, it's cheap, and it's filling. You may putty with gutta percha, you may putty with amalgam, you may putty with gold, but the worst putty of all, is cement. For getting out of trouble yourself, and getting the patient in, it beats anything in the dental pharmacopeia.

How many times have each of you had patients come from Dr. So and So, with a long story of faithful attention to the teeth, with years of inflamed gums, foul breath, and great discomfort. On examination, what is found? Putty and glue and slime. No cusps, no contours, no contacts. Gums hypertrophied, process absorbed, pus pockets, indigestion, and "visited my dentist every three months." The fact that cement dissolves away more readily, below the gum margin, makes it a menace to the hard and soft tissues. It sometimes takes years to put some cases right, and bring about a comfortable *condition.* The only remedy is hard work, well contoured inlays, good, honest, hard, well polished amalgam and sane prophylactic treatment.

Dr. Magee: Dr. Whitslar spoke of separators injuring the tissues; a great deal of injury is done by certain separators, but if any of the members here, have tried the Perry separator, and another one that has been designed on the same principle, I forget the name, they have solved a great many of the problems. I am only saying this now for those who would like to obviate the difficulties in connection with ordinary separators. The Perry 2 bar separator and the Universal Separator will separate with a modicum of injury and pain.

Perhaps, a good many will remember some years ago I undertook a bet with our old friend McInnis, that I could successfully fill the canals of seven out of ten ordinary molars that were found in practice. The teeth were handed me embeded in plaster by members of the profession, and I filled the canals and sent them to Dr. Price, of Cleveland. He made skiagraphs of them, and they were published in our Dominion Dental Journal. I lost the bet, but our worthy friend, Dr. Ottolingui, and our worthy President, both paid me a very high compliment. I want to say this, in justice to myself, that the roots were exceedingly well filled. I knew I had made a failure of three roots, I had penetrated the sides of them. I felt the conditions were hardly fair, and yet I took all the chances. Anyone who has seen the cases will acknowledge that a thorough effort was made.

In commenting on Dr. Broomell's paper, I can hardly agree with the essayist. I read it very carefully, but, with all due deference to Dr. Broomell I cannot agree with him in one of the deductions he makes. Dr. Broomell made the statement that pulps will die under a crown, and he says in his paper when you are going to adjust a crown for a bridge you should devitalize. I make it a point to save alive all pulps I can, whether it is to be used as the abutment for a bridge or to have a single crown, and I have been practicing for a good many years and I yet have to have it proved to me, that I should devitalize the pulps of teeth to be crowned. I think a tooth is very much better to be kept alive than to have it devitalized. If you are very sure you can fill that root to the end, there might be some justification for it, but no man in the world can say he can get every tooth filled to the end of the root; there may be pulp stones in it or pulp nodules. I think no man can properly claim those pulp stones are caused by irritation, resulting from caries. I have examined a great many teeth that never had a particle of caries, and I found a good many pulp stones in them.

Dr. Morrison, in his remarks, claimed that amalgam fillings should be finished at a subsequent sitting. Now, my practice is always to finish and polish the filling at one sitting. I think, the amalgam filling should not be touched below the gingival border after you have taken the rubber dam off. No cavity which projects beyond the gum border should be filled without a matrix, especially a proximal cavity, but when you finish it, you want to finish it at the one sitting. All those fillings should be made with quick setting amalgam, and there is no reason why additional time should be taken up at a subsequent sitting, for the finishing and polishing of a filling, that can more easily be done at the time of insertion. More than that, I have yet to see the nice results that will be obtained from polishing the filling afterwards, that can be obtained from polishing it at one sitting. There is a brightness that is got there, and there is a blackness which will always follow that, which will not be found if the filling is properly polished at the time it is inserted. If it is polished at the time of insertion there is a greyness that never leaves it, and it never gets quite so black as the one polished at a subsequent sitting.

Dr. Whitslar:—I agree with Dr. Morrison, that a good amalgam filling is *one* of the best fillings that can be made, and I will say to Dr. Pearson that a good cement filling, is better than a poor amalgam filling, and a good amalgam filling is better than a poor gold filling, or any inlaid filling. The great trouble to-day is, I fear, in making such cement fillings that some of us are going to offer these fillings to take the place of permanent fillings. We must treat them as temporary affairs, and talk of them as such to our patients, otherwise we will have such conditions as mentioned by Dr. Pearson. I imagine the persons he referred to as doing slovenly work are those who are slovenly in other ways, and who depend, perhaps, upon their good looks and pleasant manners for holding their practices, which is not altogether a desirable factor in a professional career. A man should have a good personal appearance, and good manners, but he should substantiate them with good work.

I also think there is a great deal in what Dr. Magee and Dr. Pearson say: I preserve the dental pulps in most teeth where I put on a crown or bridge, if they are in a healthy condition at the time. Dr. Broomell evidently seems to think that in the end the pulp will die, dry gangrene will set in, and possibly later on moisture creep in and produce conditions which will produce an abscess; but I would often rather take my chances of that, and preserve the vitality of the pulp, because, if we have that pulp in a good healthy condition, then we are more sure of the peridental membrane being healthy.

I knew that Dr. Grieve would take issue with me in regard to the extraction of the first permanent molar. I said in my paper, very rarely it should be extracted. Recently I had a case of a young girl, ten and a half years of age, whose second molars had not erupted. I found the first permanent molars very badly broken down, some of them below the gingival line. My judgment was to extract those molars and take my chances on the second molar coming into its place, and the third molar doing so normally. I referred the case to an orthodontia specialist, and he said not to extract those, but to fill and preserve the teeth. I followed the advice of the specialist, and I followed just what I have advocated in my paper, and if I am not sure of the condition myself, I go to one whose advice is superior to mine in his branch of work. The difference between a specialist in dentistry and a specialist in medicine is quite considerable. We are working upon tissues which are not repaired; our work is largely mechanical; we are not dependent upon the systemic conditions away back of us, the action of the heart and of the liver, and of the kidneys, and all those conditions; in medical science a specialist is qualified more in that line of work than a dentist would be in dental treatment.—*Dominion Journal*, June 1910.

